# Evaluating the Conservation State of Naturally Aged Paper with Raman and Luminescence Spectral Mapping: Toward a Non-Destructive Diagnostic Protocol

**DOI:** 10.3390/molecules27051712

**Published:** 2022-03-05

**Authors:** Sabina Botti, Francesca Bonfigli, Valentina Nigro, Alessandro Rufoloni, Angelo Vannozzi

**Affiliations:** 1 Physical Technologies for Safety and Health Division, Fusion and Technologies for Nuclear Safety and Security Department, Italian National Agency for New Technologies, Energy and Sustainable Economic Development (ENEA), Via E. Fermi 45, 00044 Frascati, Italy; francesca.bonfigli@enea.it (F.B.); valentina.nigro@enea.it (V.N.); 2 Superconductivity Section, Fusion and Technologies for Nuclear Safety and Security Department, Italian National Agency for New Technologies, Energy and Sustainable Economic Development (ENEA), Via E. Fermi 45, 00044 Frascati, Italy; alessandro.rufoloni@enea.it (A.R.); angelo.vannozzi@enea.it (A.V.)

**Keywords:** Raman spectroscopy, luminescence spectroscopy, cultural heritage, paper ageing diagnostics, Raman spectral imaging, luminescence spectral imaging

## Abstract

Micro-Raman and luminescence spectroscopy were combined with morphological analysis to study the conservation state of differently degraded paper samples, dated from 1873 to 2021. The aim of the work reported in this paper was to obtain ageing markers based on variations of Raman and fluorescence spectral features. Raman and luminescence spectra were acquired by scanning non-printed areas of books, and Raman and fluorescence maps were built by contrasting spectral parameters point by point, obtaining a micron-scale space resolved imaging of the degradation pattern. Complementary information on paper morphology and surface compactness were obtained by high-resolution scanning electron and atomic force microscopy. The proposed non-destructive procedure is particularly interesting for precious and ancient samples to analyze their degradation processes and to evaluate the performance and effectiveness of restoration treatments.

## 1. Introduction

The scientific approach for studying and preserving cultural heritage requires the definition of non-destructive (or minimally destructive) analytical techniques. The value of gained information about chemical–physical characterization is always balanced against the possibility of damaging or losing material of historical relevance. In this respect, intrinsically non-destructive spectroscopic techniques, such as reflection Fourier transform infrared (FTIR), Raman, and luminescence spectroscopy, have demonstrated their usefulness, particularly for library heritage, where severe restrictions in sampling are imposed for monitoring paper artwork ageing. These diagnostic techniques represent very suitable tools for the study of the interaction of paper with the environment and for the definition of appropriate treatments to prevent and hinder degradation [1,2].

The principal component of paper artwork is cellulose, which is a natural linear polymer of glucose monomers linked by glycosidic C–O–C bonds. The number of glucose monomers, indicated as polymerization degree, can be hundreds or thousands of single units, depending on the cellulose origin (wood pulp, cotton, or plant). Cellulose chains are held together by strong hydrogen bonds that promote the aggregation of single chains into a highly oriented structure, progressively forming the microfiber, the fibril, and the fiber. In cellulose, regions with regular and well-organized structure (crystalline part) and others with disordered structure (amorphous part) coexist. The ordered aggregation gives high tensile strength to cellulose fibers [3].

The ageing of paper is due to cellulose degradation, which occurs mainly through two processes: random hydrolysis of the C-O-C linkages between glucose monomers, resulting in a shortening of the cellulose chain, with a consequent reduction in content of the crystalline form, and oxidation [4,5,6]. The lower the crystallinity and polymerization degree of cellulose, the faster the deterioration, due to the higher accessibility to external agents.

Paper is produced by pressing together cellulose fibers derived from wood, rags, or grasses, and drying them into flexible sheets. In addition to cellulose fibers, paper contains hemicellulose, lignin and a certain amount of fillers that are used for bleaching and strengthening [3]. Paper made from wood pulp, as that produced at the end of the XIX century, is more readily oxidized than purely cotton paper [7]. Besides internal factors such as manufacturing, the presence of acid substances, moisture, writing media, transition metals, or micro-organisms, environmental factors such as climate, exposure to light, air pollution, and dust can affect the ageing rate of cellulose. In fact, both high levels of relative humidity and excess in temperature promote hydrolysis reactions and microbial attack. Furthermore, light causes photo-degradation phenomena, whereas the presence of suspended dust in the atmosphere increases chemical and physical damage absorbing water vapor, pollutants, and microorganisms [3].

Ageing deteriorates the mechanical and optical properties of paper material, leading to thinner and more fragile yellowed/darkened sheets, compromising the readability of the text. No significant variations in mechanical/optical properties of paper are detected at the beginning of the ageing process, whose evidence is only provided by measuring the variation of cellulose structure at molecular level. Hydrolysis reduces the cellulose polymerization degree, and oxidation leads to the carbonyl and carboxyl group formation. The recent approach of several works is to correlate these variations to the change of quantities that can be measured with non-destructive tools. In this respect, intrinsically non-destructive spectroscopic techniques gained prime importance.

In fact, the changes in the structural properties of paper due to degradation are reflected in a change of spectroscopic fingerprints values. IR, Raman, and luminescence spectroscopy were employed to study paper ageing [4,5,6,7,8,9]. Previous works demonstrated that all these techniques are able to identify degradation effects on paper exposed to artificial and natural ageing treatments. IR spectroscopy was employed in most studies for the low background contribution and simplicity of the measurement procedure. More recently, Raman spectroscopy was used to study paper ageing related to the oxidative processes by observing spectral changes in the range 1500–1900 cm^−1^ [5,6,10] in agreement with IR data.

Raman spectroscopy was also used to develop a kinetic model to date ancient papers, investigating the degradation process associated with the breaking of the glucose chain units due to hydrolysis [4,11]. In Raman spectra, the water peak at 1600 cm^−1^ is not present and does not interfere with the acquisition of the bands belonging to oxidized groups linked to cellulose backbone. The degradation of paper material causes an intensity enhancement and a profile change in the luminescence spectrum; this circumstance was used to investigate the degree of paper deterioration and estimate its manufacturing date [9].

In this respect, the aim of our work was to define a non-destructive diagnostic protocol combining Raman and luminescence spectroscopy with morphological analysis to obtain 2D chemical mapping of paper artwork conservation state across the whole page. The Raman micro-spectroscopy allows to study objects with a spatial resolution of about 1 μm, which is the same order of magnitude of a cellulose fiber diameter (0.4–20 μm). Moreover, by operating in confocal mode, only the in-focus and on-axis portion of sample contributes to the Raman signal, strongly reducing the fluorescence background. Further, surface scanning measurements allow the detection of signals at several points, taking into account the intrinsic heterogeneity of paper material.

We applied this diagnostic technique to modern and ancient paper samples to define suitable spectroscopic markers to be used as contrast parameters for building Raman and luminescence maps.

Paper samples were also analyzed with a High-Resolution Scanning Electron Microscope (HR-SEM) and an Atomic Force Microscope (AFM). SEM observations allowed us to observe a difference in the morphology between ancient and modern paper. Therefore, ageing markers were defined studying the evolution of Raman and luminescence spectra of a modern paper sample exposed to the ambient light for a period of 20 months. The as-obtained markers were used to image the conservation state of tens of different paper samples, from private library books (CC), covering a period of three centuries (XIX–XXI). The changes in the Raman spectra features can be selectively attributed to the hydrolysis and oxidation process, obtaining spectral markers that can separately monitor both the processes. Due to ageing, luminescence intensity increases with changes in shape and peak position.

The novelty of our procedure is the possibility to obtain an image with μm scale spatial resolution of paper conservation state, exploring the behavior of specific spectral markers. The proposed procedure allows us to compare the degradation processes of different paper samples, and it is of particular interest because it opens the possibility of choosing the most appropriate restoration treatment evaluating the effectiveness of the used treatment differentiating restored and non-restored regions, point-by-point, across the page. Moreover, it has been shown that this protocol, being non-destructive, can be successfully applied to precious and ancient paper samples, avoiding sampling and damaging.

## 2. Results

### 2.1. Raman and Luminescence Spectroscopy Mapping for Monitoring the Modern Paper Natural Ageing

**Figure 1:**
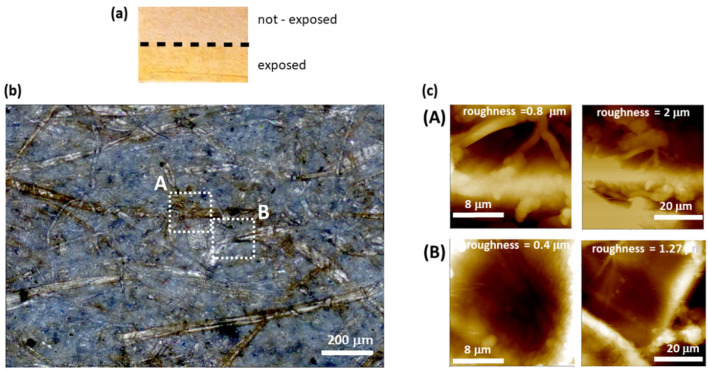
(**a**) Sketch of the portion of paper sample masked to the laboratory natural light. (**b**) Optical microscope image of the seven months exposed part of paper sample and (**c**) AFM images taken in the region (**A**) browned fiber, and (**B**) white fiber.

Before performing spectroscopic characterization of paper sample from ancient books, as the first step of our study, we exposed a piece of printer paper (2019) to natural light in the laboratory for about two years. Our aim was to follow the natural degradation evolution to define suitable contrast parameters for a proper Raman and luminescence spectral imaging of the ageing processes. A portion of paper was masked from light, as sketched in Figure 1a. Raman and luminescence maps were acquired after 3, 7, 12, and 20 months of ambient light exposition. The optical microscope image of the region of the paper sample exposed for seven months, as shown in Figure 1b, shows the browning of several cellulose fibers. The browning is not uniform, and it is particularly evident in correspondence of large fibers, likely due to inhomogeneity of the raw material. The corresponding topographic AFM images (Figure 1c) of browned (A) and white (B) fibers show that the measured roughness is determined by the type and disposition of the basic components of paper and not by the change in color.

**Figure 2:**
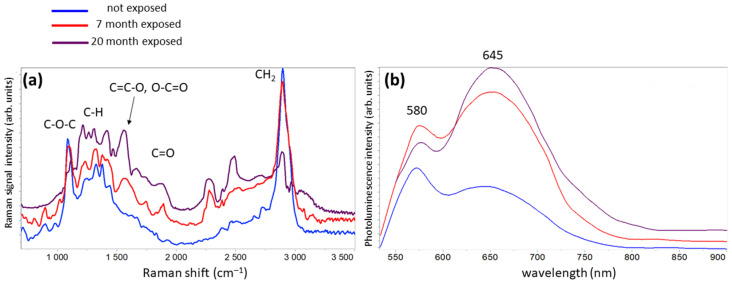
(**a**) Raman and (**b**) luminescence spectra acquired from paper sample before and after 7 and 20 months of exposition.

Figure 2 reports both Raman and photoluminescence spectra acquired from the paper sample before and after 7 and 20 months of exposition. The main peaks observed in Raman traces of Figure 2a can be related to vibrational features of cellulose carbon groups, namely the stretching of C–O–C glycosidic bonds between cellulose monomers (1100 cm^−1^), the internal vibrations of the C–H groups (1300–1470 cm^−1^) and the CH_2_ stretching peak, which is related to the crystalline degree of cellulose (2890 cm^−1^). The fingerprint at 1602 cm^−1^ showing the presence of lignin was not observed in this sample of laser printer paper. The exposition to ambient light causes intensity decrease and broadening of C–O–C and CH_2_ peaks and the appearance of new bands, whose intensity increases with exposition time, related to the oxidized species attached to cellulose chain, namely the bands at 1580 cm^−1^ (stretching of double bonds in C=C–O and O–C=O groups), 1640 cm^−1^, 1740 cm^−1^ (stretching of C=O in the carbonyl), 1850 cm^−1^ (stretching of C=O in the carboxyl groups), and in the 2300–2800 cm^−1^ region (overtones and combination of carboxylic group frequencies) [5,7,8].

As paper ageing proceeds, the length of cellulose polymer chain decreases due to hydrolysis reactions. This process can be monitored by recording the intensity of the bands at 1100 and 1380 cm^−1^. The intensity of the first band, ascribed to the stretching of the C–O–C glycosidic bond, is proportional to the number of linkages in the cellulose chain, while the intensity of the second band, related to the vibrations of C–H groups in the glucose monomer, is weakly sensitive to the cellulose polymerization degree. Thus, the intensity ratio *R*_H_ = *I*_1100_/*I*_1380_ calculated from the measured Raman spectra is proportional to the polymerization degree of cellulose in the paper sample [4]. Indeed, the *R*_H_ marker decreases as the ageing/exposition time of the paper sample increases. Analogously, the index *CI* = *I*_2890_*/I*_1380_ of cellulose crystallinity describes the decrease in CH_2_ peak intensity due to the reduction in content of crystalline form, consequent to the shortening of cellulose chain length in exposed paper.

The modification in cellulose chain induced by oxidation can be evaluated from the strength of new peaks appearing in the Raman spectra of exposed papers. The oxidation of hydroxyl groups in a cellulose chain in an ambient atmosphere leads to the formation of both C=C double bonds in glucose ring and of C=O bonds (carbonyl groups), which can be further oxidized to carboxylic groups, depending on the position of the C=O bonds in cellulose chain [5,8,12]. The ratio *O*_I_ = *A*_1640__–1850_/*A*_1580_ between the area of carbonyl bands in the range 1640–1850 cm^−1^ (*A*_1640__–1850_), representing the final oxidation stage of cellulose, and that of the band at 1580 cm^−1^ (*A*_1580_), corresponding to the presence of intermediate species, gives information on how advanced the oxidation process is [5,10]. In contrast, the ratio *O*_T_ = *A*_1500–2800_/*A*_700__–3000_ between the area of Raman bands of oxidized functional groups in the range 1500–2800 cm^−1^ (*A*_1500__–2800_) and the area of whole spectrum (*A*_700__–3000_) measures the amount of oxidation products linked to the cellulose backbone.

Figure 2b reports the photoluminescence spectra of the same paper samples of Figure 2a, exhibiting two broad bands centered at 580 and 650 nm. By increasing the exposition time, the total area of luminescence spectrum increases, while the intensity ratio I_R_ between the first and second peak decreases. Due to the similarity in shape between the 580 nm peak and the one measured for the wood cellulose, with two peaks (one centered at 488 nm and the other at 580 nm), recent works correlate the presence of the long cellulose chains in paper to lower wavelength peak in the explored spectral region [13,14]. Following this hypothesis, fiber fragmentation promoted by hydrolysis causes an intensity decrease in the first peak. The increase in luminescence intensity of the second peak is ascribed to the formation of compounds originating from the degradation of cellulose, hemicellulose, and lignin as simple sugars, cellulose oligomers, and phenolic products.

**Figure 3:**
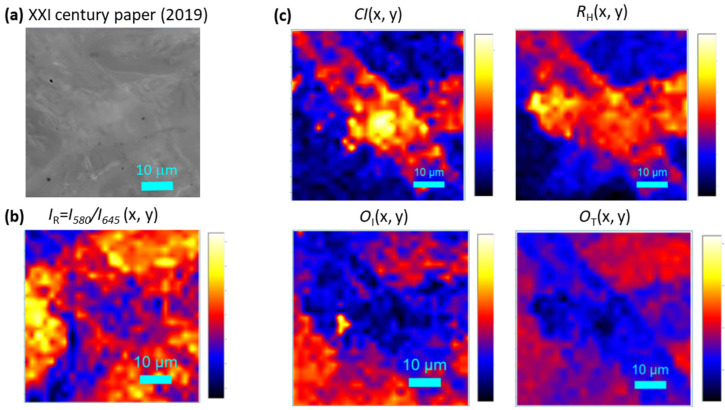
(**a**) White light optical image (objective 100×) of 60 × 60 μm^2^ of non-exposed paper sample area selected for spectra acquisition. Maps from 2D spectral arrays of (30 × 30) complete Raman/luminescence spectra recorded in the spectral imaging mode and extracted using as contrast parameters (**b**) the ratio *I*_R_ between the luminescence peak intensities and (**c**) the *CI*, *R*_H_, *O*_I_ and *O*_T_ markers from Raman spectra. The color code displays the increasing value in the sequence blue–red–yellow.

Figure 3a shows the white light optical image of non-exposed paper sample. The area was scanned in Raman/photoluminescence spectral imaging mode, and 2D spectral arrays of (30 × 30) complete Raman/luminescence spectra were recorded. From these 2D spectral arrays, five distinct maps for cellulose characterization were extracted, using as contrast parameters the ratio *I*_R_ = *I*_580_/*I*_645_ between the intensities of the luminescence peaks (Figure 3b) and the *CI*, *R*_H_, *O*_I,_ and *O*_T_ markers calculated from Raman spectra (Figure 3c).

**Figure 4:**
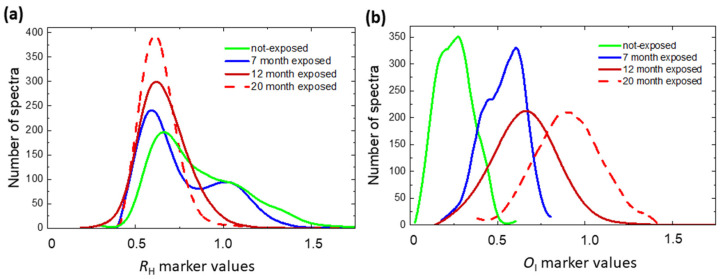
Distributions of (**a**) *R*_H_ and (**b**) *O*_I_ values extracted from the maps reported in Figure 3c before and after 7, 12, and 20 months of exposure of paper sample.

These marker maps are referred to the same area of crossing fibers shown in the white light image of optical microscope (Figure 3a). The used color code displays the increasing value in the sequence blue–red–yellow. Larger values of *CI* and R_H_ markers, measured along the cellulose fiber wall, are correlated with smaller values of *O*_I_ and *O*_T_ markers. This finding gives rise to a bimodal distribution of *R*_H_ and *O*_I_ values, extracted from the map of Figure 3c, as shown in Figure 4. By increasing the exposition time, the *R*_H_ (*O*_I_) distribution peak decreases (increases) as expected, and the shape of distributions changes, becoming unimodal.

**Figure 5:**
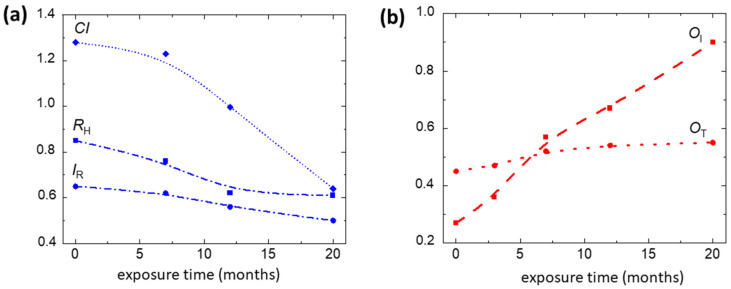
Behavior of the mean values of the (**a**) *I*_R_, *CI*, and *R*_H_ and (**b**) *O*_I_ and *O*_T_ markers, averaged over the distribution, as a function of the exposure time.

Figure 5 reports the mean values of markers, averaged over the distribution, as a function of exposition time. The decrease in crystalline index (*CI*) is accompanied by a decrease in both polymerization degree (*R*_H_) and 580 nm luminescence peak intensity (*I*_R_), related to the long cellulose fiber. *I*_R_ and *R*_H_ markers show an initial rapid decrease followed by a slower variation, whereas the first stage rapid increase in *O*_T_ marker, proportional to the content of oxidized groups in the cellulose backbone, saturates after 12 months of exposure (Supplementary Material S2). In contrast, the continuous increase in *O*_I_ marker indicates a progressive advancement of oxidation state with the production of C=O double bonds.

This behaviour agrees with literature data, which report that cellulose degradation can be regarded as taking place in three or two steps [3,5,15]. In the first rapid stage, the weak or acid sensitive links are firstly hydrolysed, whereas in the second one, only the amorphous part of cellulose is randomly hydrolysed, as shown by the bimodal distribution of ageing marker values. At this stage, the ageing reaction decelerates, and the crystalline part of cellulose is attacked by hydrolysis/oxidation processes. Thereon, the ageing proceeds slowly and homogeneously, and the ageing marker value distributions are unimodal. It is worth noticing that, opposed to what happens for the *O*_T_ index, the *O*_I_ marker increase does not saturate, suggesting a continuous transformation of the intermediate products of oxidation into carbonyl groups.

### 2.2. Raman and Luminescence Spectroscopy Mapping of Modern and Ancient Paper

**Figure 6:**
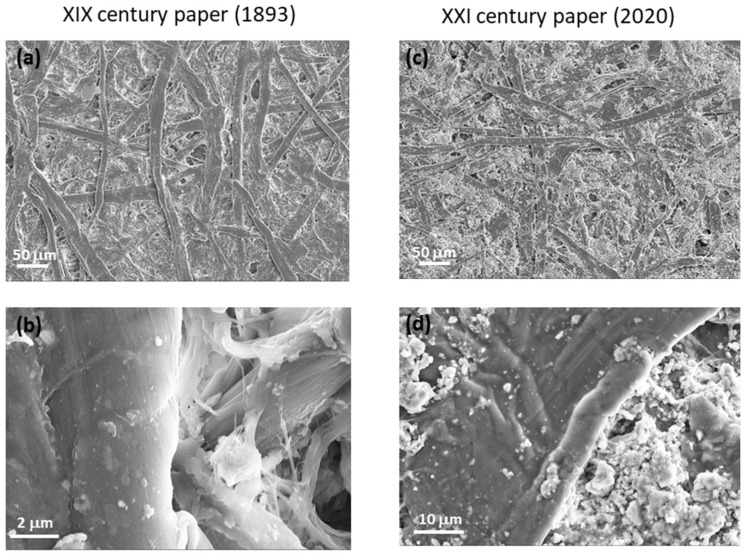
HR-SEM images of two paper samples of (**a**,**b**) XIX and (**c**,**d**) XXI century at different magnifications.

Figure 6 shows HR-SEM images of two paper samples of XIX (Figure 6a,b) and XXI (Figure 6c,d) centuries. Long cellulose fibers are clearly visible in the pictures related to the XIX century paper sample, whereas in those of modern paper, the fibers are mixed with the presence of granular material. This finding demonstrated the change in paper composition over the centuries. In fact, modern paper is composed of only up to 50% short cellulosic fibers, the remaining part being hemicellulose and lignin. On the other hand, ancient paper was manufactured from long cellulose fibers in larger percentages with respect to the hemicellulose/lignin content [7,9].

**Figure 7:**
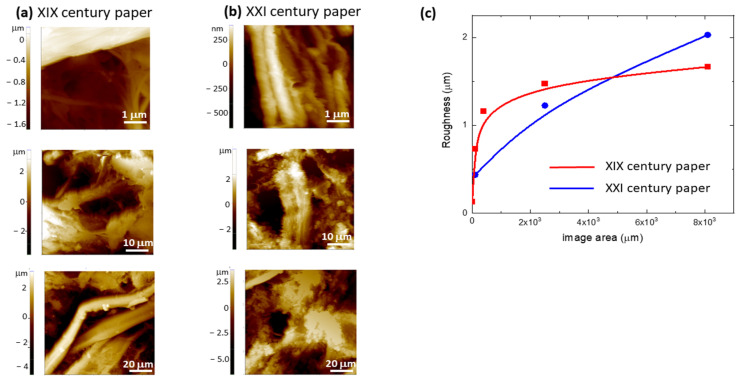
AFM images acquired on (**a**) XIX and (**b**) XXI century paper by scanning areas of different size. (**c**) Values of the local roughness calculated as standard deviation from the average value of the height histogram built on each area for XIX and XXI century paper.

Several topographic AFM images were acquired on ancient and modern papers by scanning areas of different size, as shown in Figure 7a,b. The AFM images show that the cellulose fibers of both samples are in a good conservation state without visible breakages, at least in the analyzed regions, and demonstrate the presence of disordered material in between the fibers. For each area, the height histogram was drawn, and the corresponding local roughness was calculated as standard deviation from average value. The obtained values are reported in the plot of Figure 7c. By increasing the AFM image area, the roughness of ancient paper increases and saturates at 1.7 μm, whereas modern paper roughness progressively increases with any saturation observed.

This behavior can be ascribed to the more homogeneous composition of ancient paper with respect to modern paper, as evidenced in the HR-SEM images. The literature data report that paper ageing causes a lack of fiber surface compactness [12]; instead, in this experiment, the roughness of modern paper is larger than that of ancient paper due to its inhomogeneous composition and its short cellulosic fibers content.

**Figure 8:**
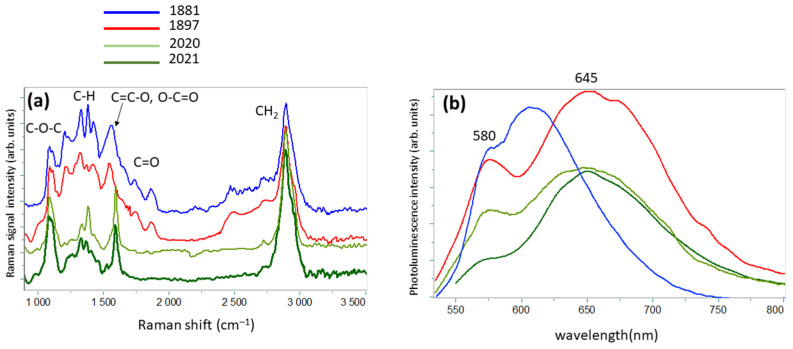
(**a**) Raman and (**b**) luminescence spectra from XIX century paper compared with those of modern paper.

Figure 8a reports representative Raman spectra from XIX century paper compared with those of modern paper. The measured Raman profiles of ancient paper are quite similar to those collected from exposed paper, with oxidized group bands above 1500 cm^−1^, whereas in the Raman spectra of modern paper, a lignin peak at 1600 cm^−1^ is present. The use of wood pulp rather than rags causes an increase in lignin content in paper that can be eliminated only with secondary long chemical processes using toxic substances. Today, it is preferred not to eliminate lignin, leading to the production of low-cost, low-quality paper [16]. Therefore, the analyzed modern paper samples were manufactured from short cellulose fibers as the main component (see Figure 6b), but also with lignin and hemicellulose as secondary components. This difference in manufacturing is reflected in the value of I_R_ ratio from the luminescence spectra reported in Figure 8b, which is smaller in modern than in ancient paper. Therefore, this parameter cannot be used to estimate the degradation degree of paper in a direct and simple way. However, the wavelength distance between the two luminescence peaks clearly shortens with increasing paper age. In fact, a shift of wavelength peak (w_p_) of smaller energy band toward the 580 nm peak is observed.

**Figure 9:**
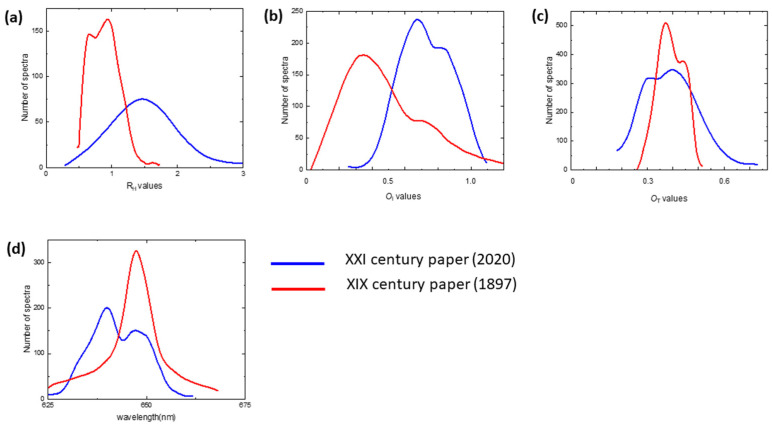
Distributions of (**a**) *R*_H_, (**b**) *O*_I_ and (**c**) *O*_T_ values from Raman spectra together with (**d**) the distribution of wp from luminescence spectra for XXI and XIX century paper.

We acquired Raman and luminescence spectra from an area of 60 μm × 60 μm for XIX and XXI century paper samples. The distribution of marker values calculated from Raman spectra as defined in the previous paragraph are reported in Figure 9a–c, together with the distribution of w_p_ obtained from the measured luminescence spectra (Figure 9d).

Modern paper displays a long tail of *R*_H_ values larger than those of ancient paper, which experienced 124 years of hydrolysis attack. The *O*_I_ distribution of ancient paper peaked at about 0.65 and 0.85, whereas that of modern paper peaked at 0.3, with a shoulder at 0.7. The *O*_T_ values peak is at about the same position for both paper samples, with a larger width for modern paper. As expected from the above discussion, in the ancient paper, oxidation proceeds homogeneously and is more advanced, with a larger content of carbonyl groups, whereas the increase in hemicellulose and lignin contents, which are easier to oxidize, accelerates the degradation rate of modern paper.

As shown in Figure 9d, the w_p_ distribution in ancient paper samples shows two peaks, one centered at about the same value of modern paper and the other one at 640 nm. The first peak may be due to the permanence of long cellulose fiber in the ancient paper sample, whereas the shifted peak is associated with changes occurred in paper structure related to the more rapid denaturation of shorter chains and to other bio-polymeric compounds present in the paper [13,17]. 

**Figure 10:**
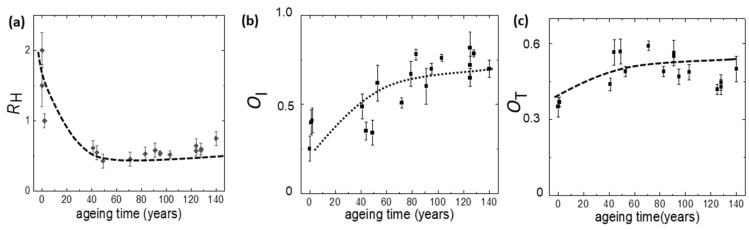
Behavior of the mean values of the (**a**) *R*_H_, (**b**) *O*_I,_ and (**c**) *O*_T_ markers from Raman spectra of several regions of non-printed areas of books of XIX, XX, and XXI centuries as a function of the ageing time.

Non-printed areas of books of XIX, XX, and XXI centuries were analyzed. For each paper sample, several regions were mapped (Supplementary Material S3), obtaining the distributions of different marker values. Figure 10 plots the evolution of *R*_H_, *O*_I,_ and *O*_T_ markers with ageing time. The reported values are the mean values calculated over the distributions, and the error bars are the calculated standard deviations. The observed difference in the paper manufacturing accounts for the large *O*_T_ values of modern paper with respect to those of XIX century paper, in which degradation is slow. The *O*_I_ values increase monotonically with ageing time, showing several fluctuations that can be explained with the different storage condition experienced by the books from which the sample paper is taken. In this respect, the *R*_H_ marker shows a steep decrease with ageing time, although the initial values of XXI century paper samples are scattered.

## 3. Materials and Methods

We studied several paper samples from books belonging to library heritage of our families and covering a period of three centuries (XIX–XXI). The paper ages are certified by publication dates. We analysed from each book at least four different areas, collecting Raman and luminescence spectra from non-printed areas mainly located at the page edges.

A Zeiss Z2m optical microscope was used for preliminary sample observation.

Raman and luminescence spectra were acquired with a confocal micro-Raman spectrometer (Horiba XploRA Plus) with a 532 nm-wavelength laser. The Raman/luminescence signals were collected through microscope equipped with 5×, 10×, 50×, and 100× objectives. Laser power can be attenuated by neutral density filters. After preliminary studies, suitable conditions for both laser power and acquisition times were selected to obtain a good S/N ratio and safe conditions for the paper. Raman and luminescence spectra were recorded at every point of a selected area following a prefixed grid defining a 2D map and building a 2D array of spectra. The recorded spectra were background subtracted and smoothed. Areas ranging from 0.0036 to 0.64 mm^2^ were scanned with different grid step size. By extracting Raman/luminescence spectra features, such as peak intensity, band width, and peak position, from the recorded 2D arrays of spectra, different spectral images could be obtained.

The spectral maps reported in this paper were obtained under the following conditions: laser power 25 mW, accumulation time 0.7 s, range 70–4000 cm^−1^, for the Raman spectra, and laser power 1 mW, accumulation time 1 s, for the luminescence spectra. In both cases, the 2D array contained 900 spectra, acquired with an objective 100× in an area of 60 × 60 μm^2^, with a x,y grid step size of 2 μm.

The morphology of modern and ancient paper was examined by using a field-emission Zeiss HR-SEM Leo 1525 with a resolution of 1.5 nm at 20 kV.

We also performed surface analysis using a Park Systems XE-150 AFM operating in non-contact mode. Pre-mounted non-contact, high-resolution cantilevers working at 309 MHz with nominal tip radius below 10 nm were used. Images were flattened by subtracting a linear background for the fast scan direction and a quadratic background for the slow scan direction. Different paper sample regions were analyzed, extracting surface height histogram and the corresponding local surface roughness defined as the standard deviation of surface height with respect to the mean value.

## 4. Conclusions

In this work, we followed the natural degradation evolution of paper exposed to natural light to define suitable contrast parameters for the spectral imaging of ageing processes. In this way, we were able to define a procedure for performing spectroscopic characterization of paper sample from ancient books covering a period of three centuries, from 1873 to 2021.

The proposed diagnostic protocol is based on Raman and luminescence spectroscopy combined with morphological analysis to obtain 2D chemical mapping of paper artwork conservation state across the whole page and, being non-destructive, is of interest for conservation and restoration of precious and ancient books and papers.

Paper samples of different ages undergo different degradation processes; moreover, for each paper sample, different ageing patterns can be observed. In spite of this difficulty, once we defined the correct spectral markers to be used as contrast parameters in the Raman and luminescence maps, the spectral imaging offered a suitable description of such patterns, across the sampled area, with different length scales.

However, the number of fingerprint peaks in Raman spectra is larger than those of luminescence spectra. In particular, the markers obtained from Raman spectra can discriminate between hydrolysis and oxidation processes, whereas a deconvolution should be applied to the luminescence spectra to discriminate the different contributions of paper degradation, making the analysis of paper ageing based on this spectroscopic technique more complicated.

The obtained results confirmed that the applied method can be used to compare degradation processes of different paper samples in a quick and reliable way.

The possibility to successfully apply this method for evaluating the effectiveness of cleaning and restoration treatment on paper artifacts will be the subject of our next work.

## Data Availability

Not applicable.

## Supplementary Materials

The following supporting information can be downloaded online. S1: pictures of analysed books; S2: Raman maps of *O*_T_ marker for not-exposed and exposed paper; S3: Raman maps of *R*_H_, *O*_I,_ and *O*_T_ markers for XIX century paper.

## Sample Availability

Paper samples are available from the authors.
