# Supplementary material for: Evaluating the Conservation State of Naturally Aged Paper with Raman and Luminescence Spectral Mapping: Toward a Non-Destructive Diagnostic Protocol
Source: Molecules. 2022 Mar 5;27(5):1712. doi: 10.3390/molecules27051712 (PMC8911975; doi:10.3390/molecules27051712)

Supplementary materials S3

Raman mapping of R_H, OI and O_{tot} markers

(60 μm x 60 μm, 2 μm grid step size, 900 spectra)

PAPER OF XIX century

Raman mapping of R_H, OI and O_{tot} markers (60 µm x 60 µm, 2 µm grid step size, 900 spectra)
PAPER OF XIX century

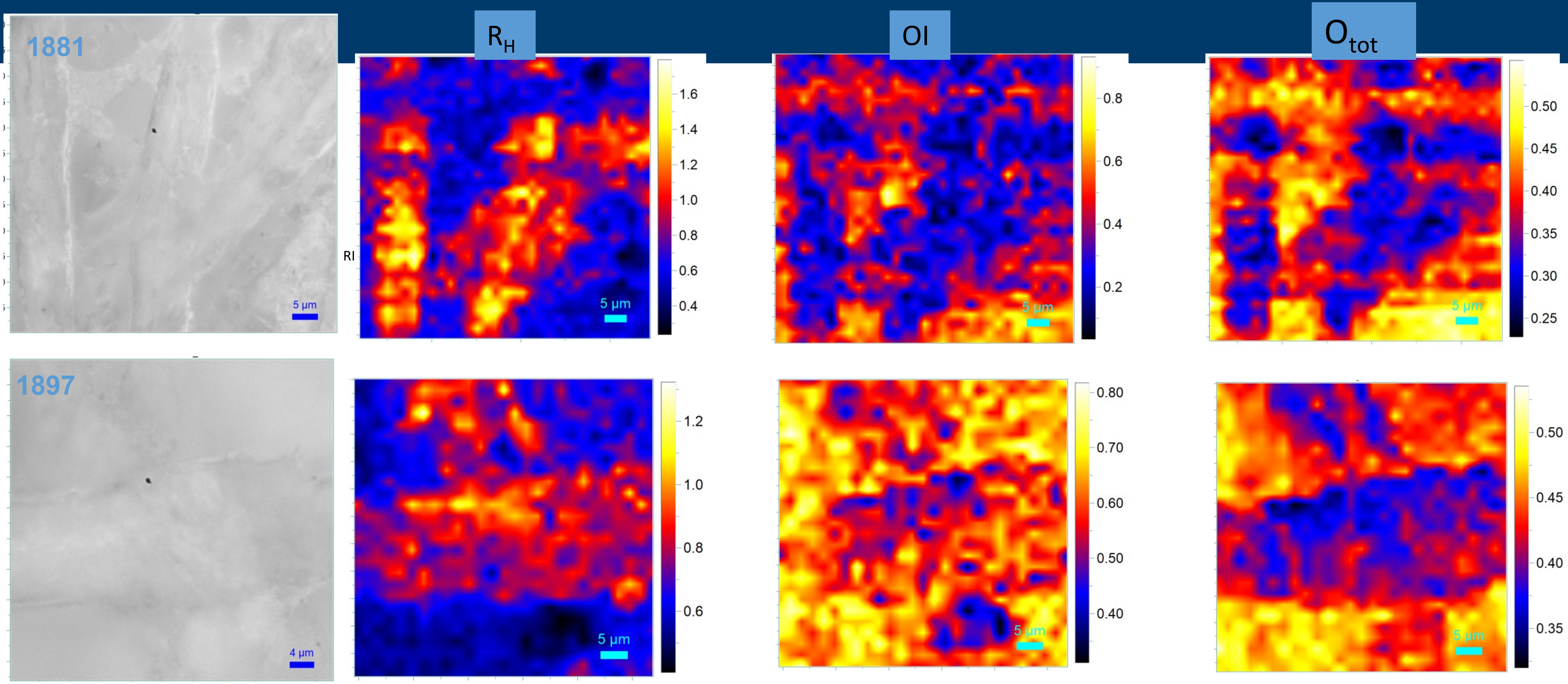

Supplement: Supplementary file 1 [file molecules-27-01712-s001.zip › supplementary_materials/Supplementary materials S3.pdf]
