# Supplementary material for: Evaluating the Conservation State of Naturally Aged Paper with Raman and Luminescence Spectral Mapping: Toward a Non-Destructive Diagnostic Protocol
Source: Molecules. 2022 Mar 5;27(5):1712. doi: 10.3390/molecules27051712 (PMC8911975; doi:10.3390/molecules27051712)

Vita degli Animali, vol.6, Brehm, 1896 (S. Botti private library)

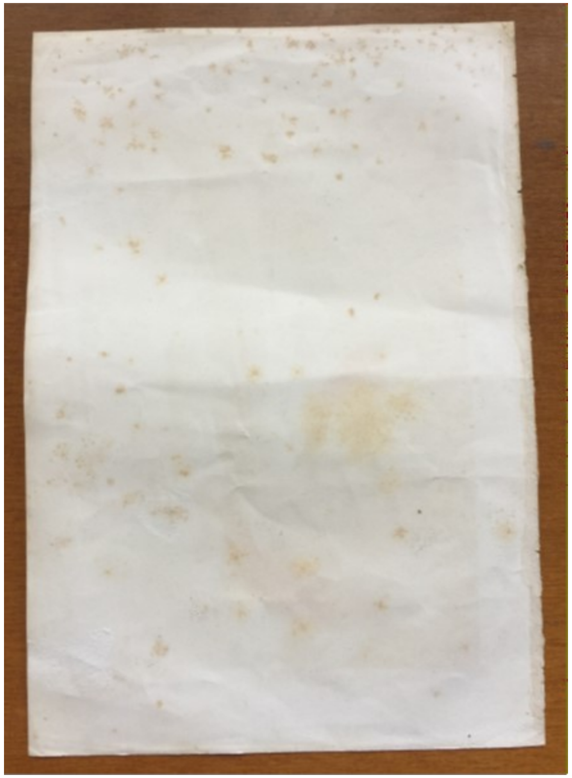

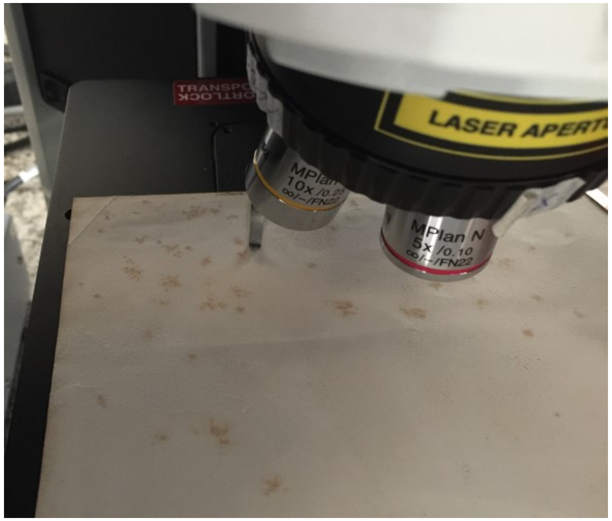

Sulla Riproduzione del diamante, Q. Majorana, 1897 (V. Nigro private library)

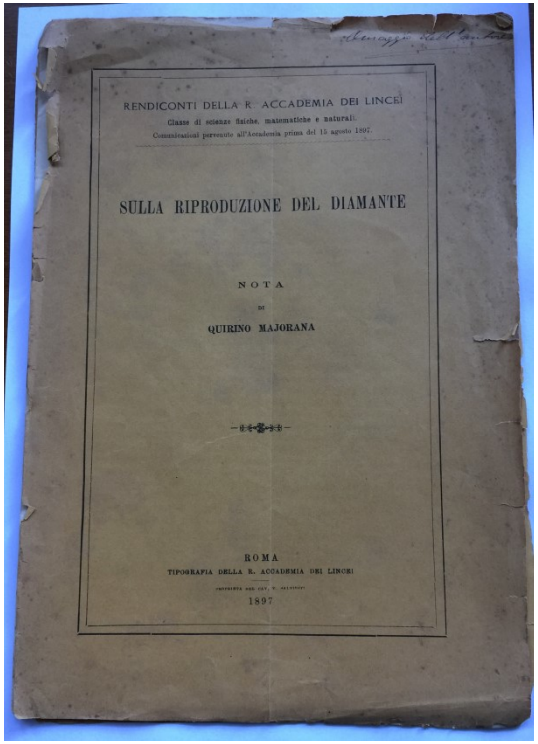
RENDICONTI DELLA R. ACCADEMIA DEI LINCEI
Classe di scienze fisiche, matematiche e naturali.
Comunicazioni pervenute all'Accademia prima del 15 agosto 1897.

SULLA RIPRODUZIONE DEL DIAMANTE

NOTA
DI
QUIRINO MAJORANA

ROMA
TIPOGRAFIA DELLA R. ACCADEMIA DEI LINCEI
[illegible]
1897

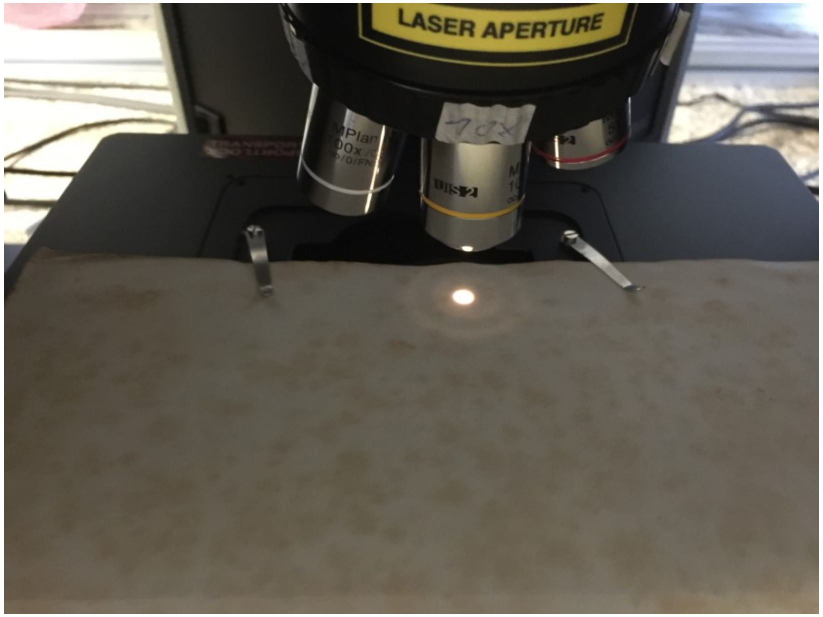

Tables de Logarithmes a cinque décimal, J. Dupuis, Librairie Hachettes, Paris, 1918 (S. Botti private library)

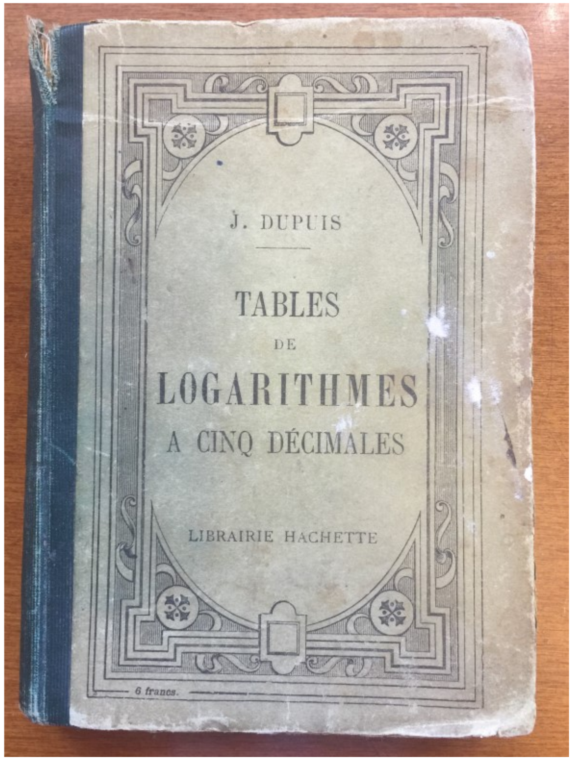

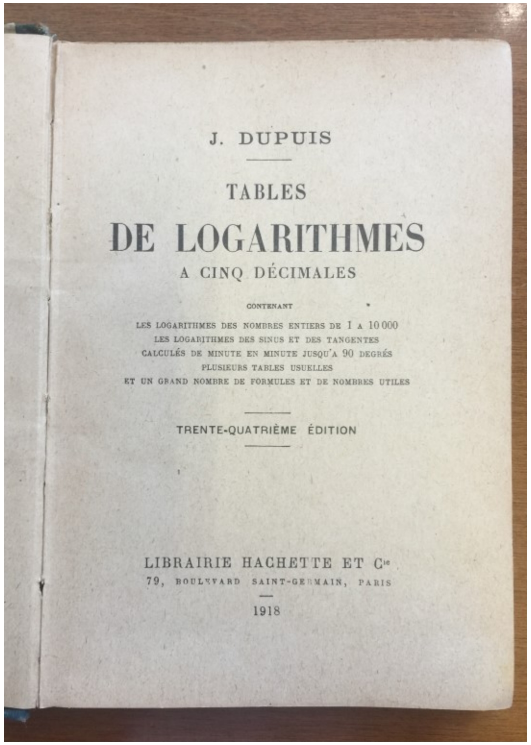

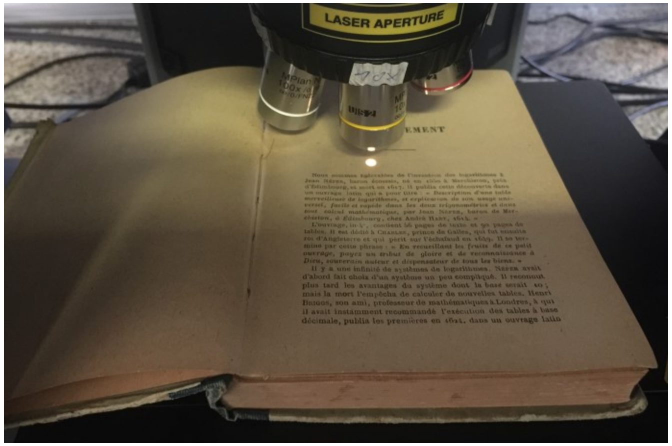

Problemi di Matematica elementare – Alfredo Bassi, Raffaello Giusti Ed., 1926
(S. Botti private library)

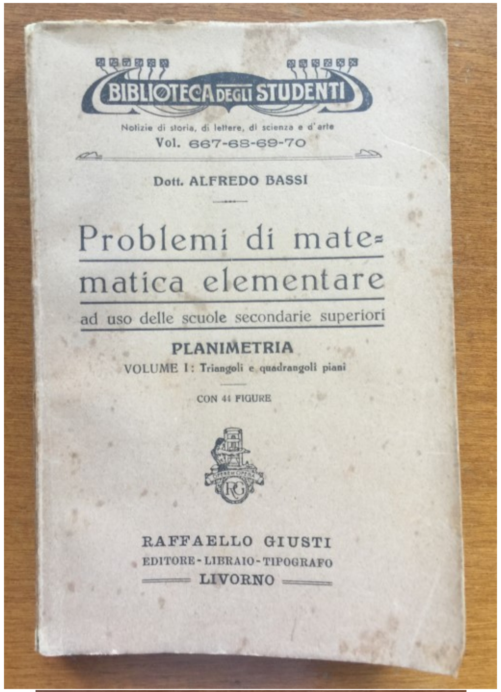

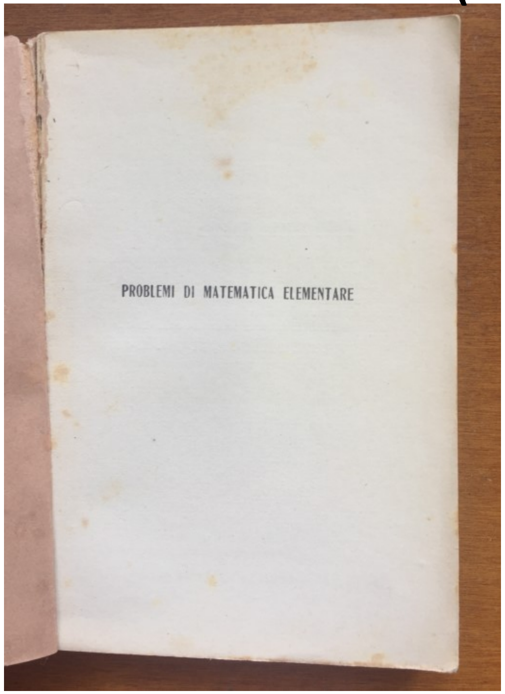

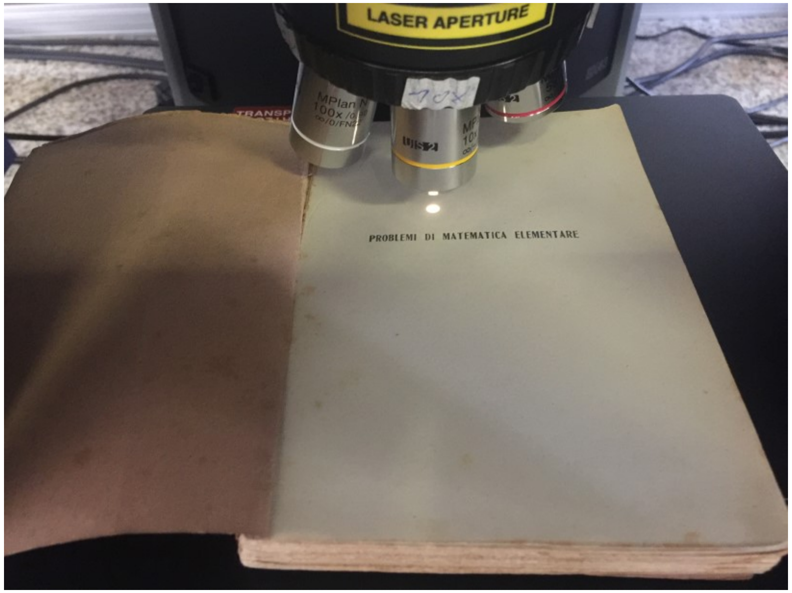

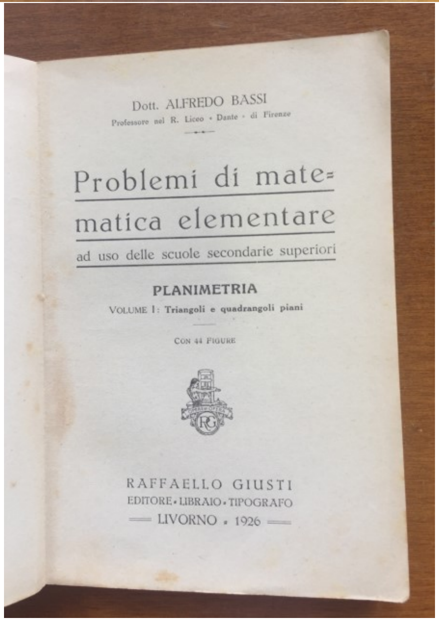

Corso di Trigonometria, A. Falanga, 1943 (S. Botti private library)

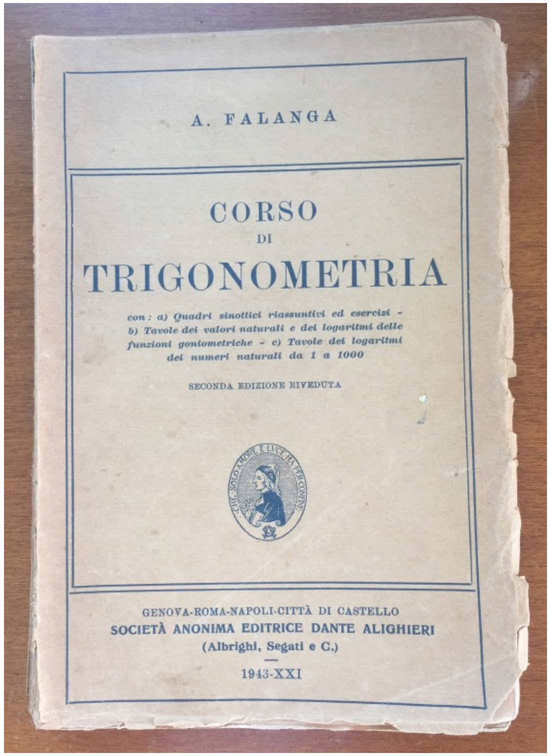

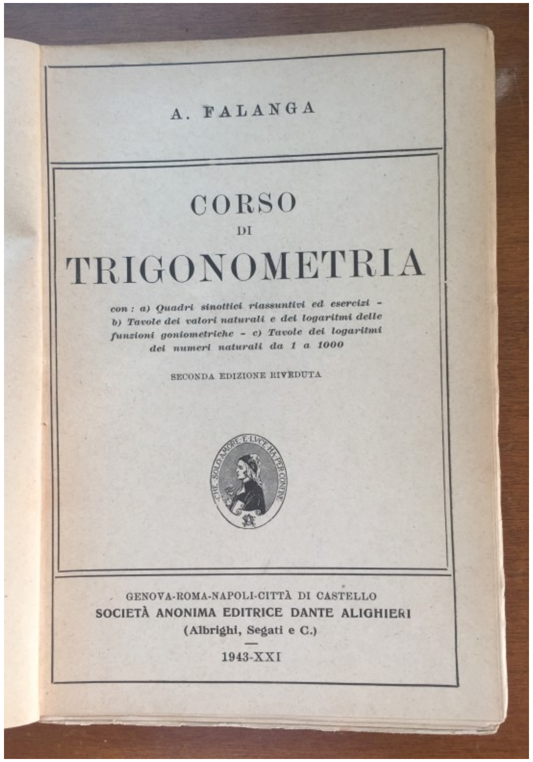

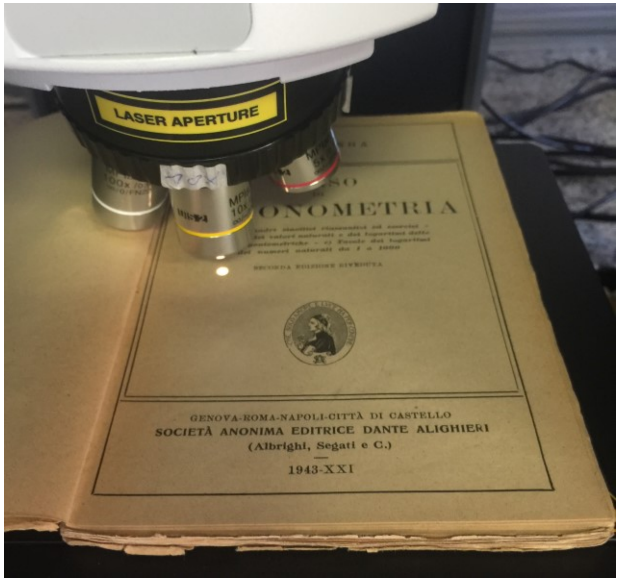

Bacie e Spari, Hugo Prat, 1973 (Courtesy of E. Giovenale)

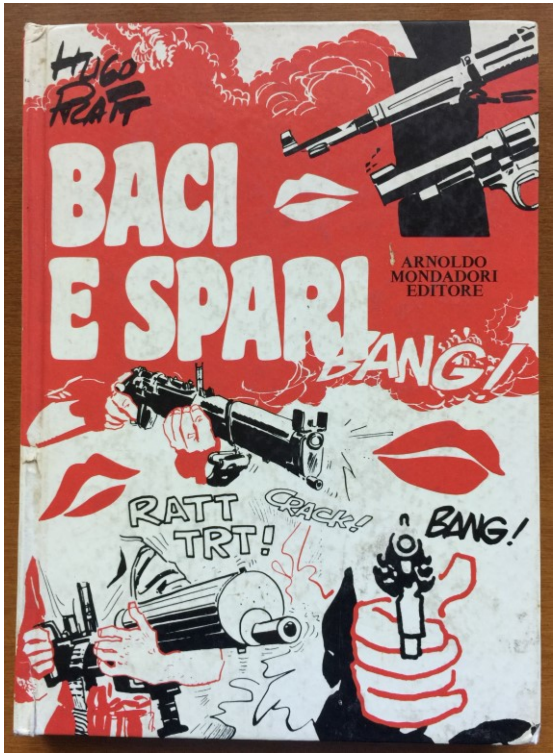

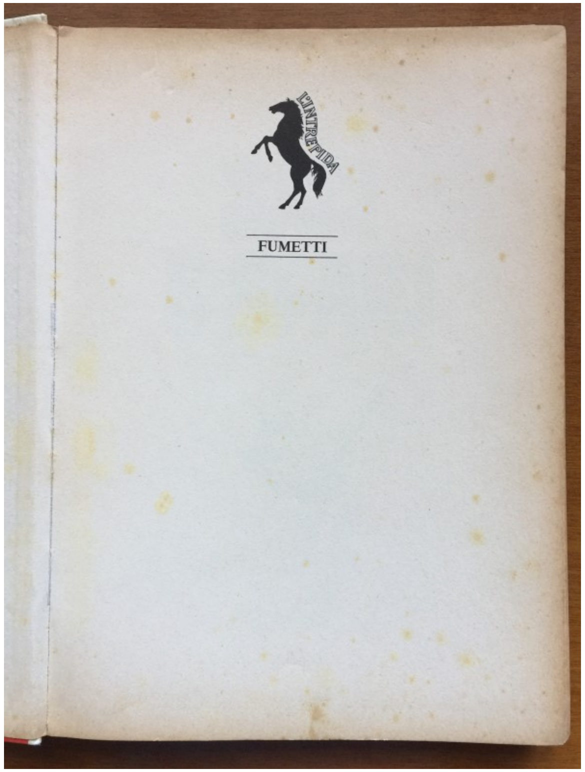

Un maggiordomo nel Far West, S. Fleischman, Oscar Ragazzi Mondatori, 1972 (Courtesy of E. Giovenale)

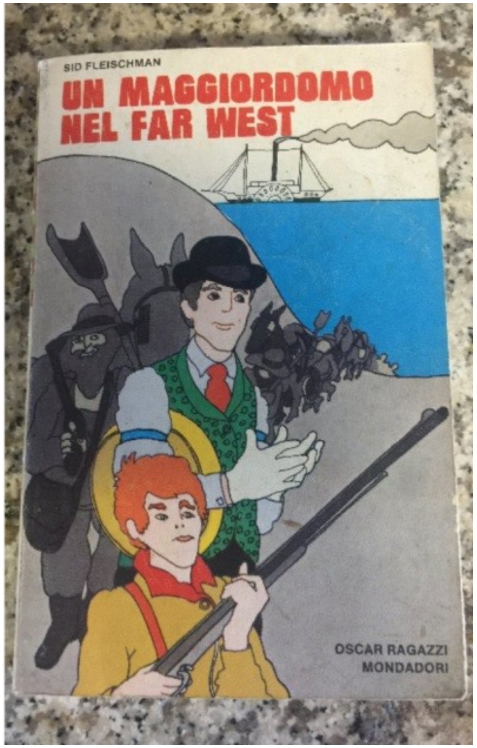

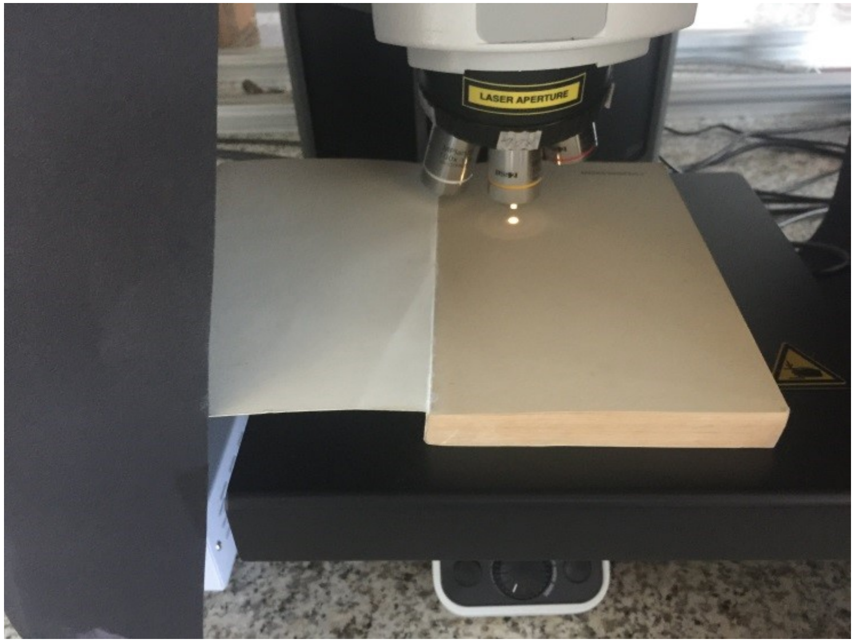

Exposed modern paper

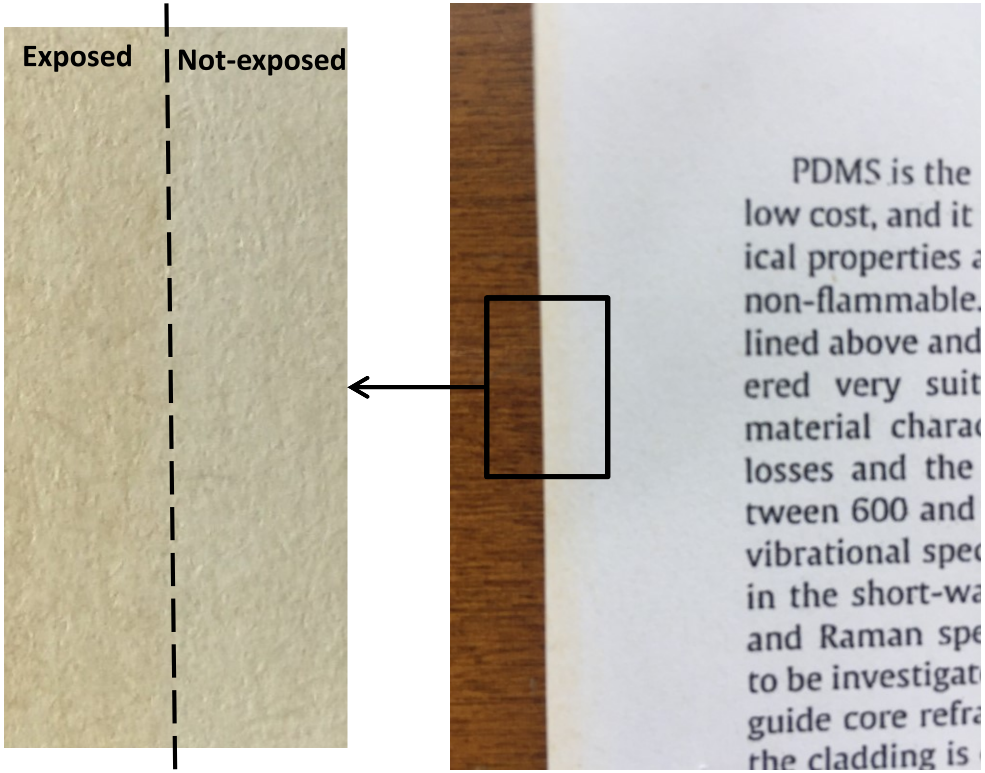

Supplement: Supplementary file 1 [file molecules-27-01712-s001.zip › supplementary_materials/Supplementary_material_S1.pdf]
