# Supplementary material for: Evaluating the Conservation State of Naturally Aged Paper with Raman and Luminescence Spectral Mapping: Toward a Non-Destructive Diagnostic Protocol
Source: Molecules. 2022 Mar 5;27(5):1712. doi: 10.3390/molecules27051712 (PMC8911975; doi:10.3390/molecules27051712)
Supplement: Supplementary file 1 [file molecules-27-01712-s001.zip › supplementary_materials/Supplementary_material_S2.pdf]

Supplementary materials_S2

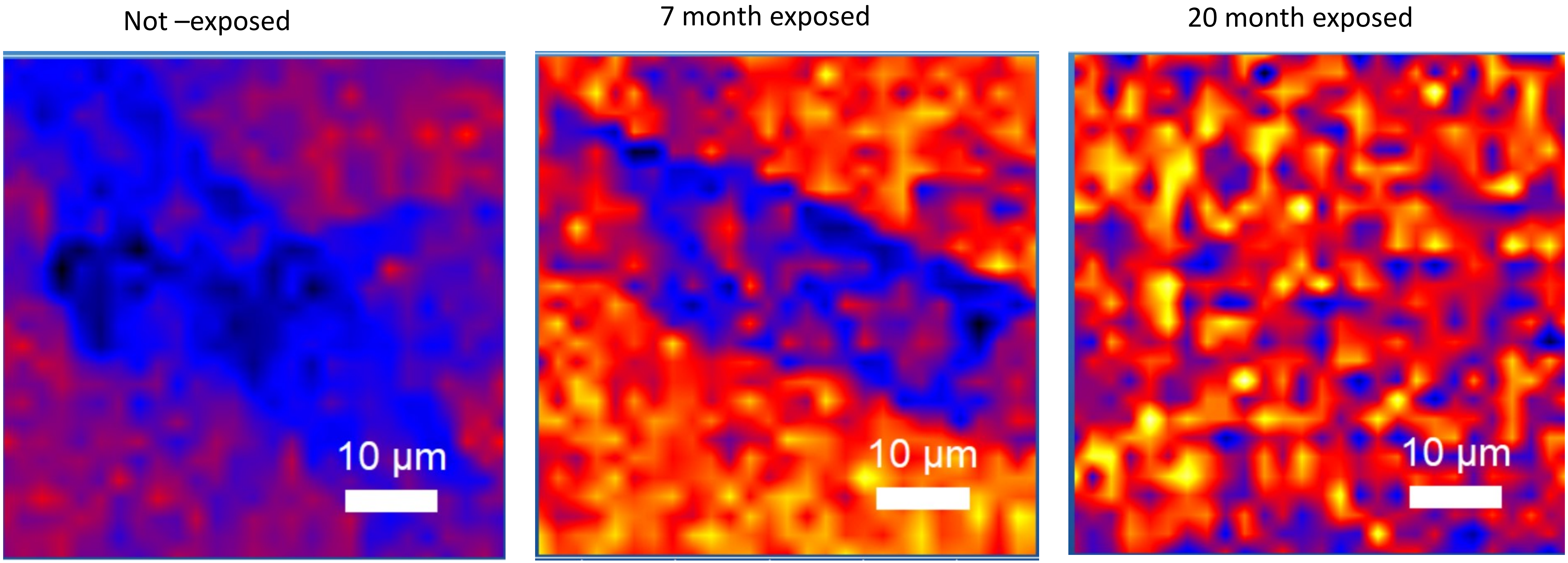

O_T Raman maps for exposed and not- exposed paper
